# Presep: Predicting the Propensity of a Protein Being Secreted into the Supernatant when Expressed in *Pichia pastoris*

**DOI:** 10.1371/journal.pone.0079749

**Published:** 2013-11-21

**Authors:** Jian Tian, Yuhong Zhang, Bo Liu, Dongyang Zuo, Tao Jiang, Jun Guo, Wei Zhang, Ningfeng Wu, Yunliu Fan

**Affiliations:** 1 Biotechnology Research Institute, Chinese Academy of Agricultural Sciences, Beijing, China; 2 Key Laboratory of Agricultural Genomics (Beijing), Ministry of Agriculture, Beijing, China; Russian Academy of Sciences, Institute for Biological Instrumentation, Russian Federation

## Abstract

*Pichia pastoris* is commonly used for the production of recombinant proteins due to its preferential secretion of recombinant proteins, resulting in lower production costs and increased yields of target proteins. However, not all recombinant proteins can be successfully secreted in *P. pastoris*. A computational method that predicts the likelihood of a protein being secreted into the supernatant would be of considerable value; however, to the best of our knowledge, no such tool has yet been developed. We present a machine-learning approach called Presep to assess the likelihood of a recombinant protein being secreted by *P. pastoris* based on its pseudo amino acid composition (PseAA). Using a 20-fold cross validation, Presep demonstrated a high degree of accuracy, with Matthews correlation coefficient (MCC) and overall accuracy (Q2) scores of 0.78 and 95%, respectively. Computational results were validated experimentally, with six β-galactosidase genes expressed in *P. pastoris* strain GS115 to verify Presep model predictions. A strong correlation (R^2^ = 0.967) was observed between Presep prediction secretion propensity and the experimental secretion percentage. Together, these results demonstrate the ability of the Presep model for predicting the secretion propensity of *P. pastoris* for a given protein. This model may serve as a valuable tool for determining the utility of *P. pastoris* as a host organism prior to initiating biological experiments. The Presep prediction tool can be freely downloaded at http://www.mobioinfor.cn/Presep.

## Introduction

*Pichia pastoris* is one of the most frequently used organisms for the heterologous production of recombinant proteins. It is well-characterised, easy to manipulate genetically, requires minimal safety precautions, and can be grown quickly and inexpensively to high cell densities [1], [2]. In addition, the majority of recombinant proteins expressed in this organism are secreted directly into the culture medium. This preferential secretion of recombinant proteins allows for direct isolation of target proteins from culture media, eliminating the need for high-cost, low-yield cell disruption. Furthermore, this feature limits toxicity issues resulting from intracellular accumulation of target proteins. However, not all recombinant proteins can be successfully secreted in yeast, and the intracellular retention of some highly expressed proteins is still a problem, limiting more widespread use.

A variety of methods have been developed to enhance the secretion of recombinant proteins in *P. pastoris*. Studies have shown that increases in gene dosage [1], changes to promoters or signal sequences [3], and co-overexpression of molecular chaperones [4], [5], [6], protein disulfide isomerase (PDI) [7], [8] and unfolded protein response factor (UPR) [9] can enhance the secretion of some recombinant proteins. While the efficacy of these methods has been demonstrated for a variety of proteins, they may not be sufficient for proteins not normally secreted by the original cell. Therefore, attempts to express such proteins in *P. pastoris* may consume significant time and resources, with no way to predict the likelihood of success. A method that predicts the likelihood of a protein being secreted into the supernatant before being expressed in *P. pastoris* would be of considerable value; however, to the best of our knowledge, no such tool has yet been developed.

Secretion signals have recently been shown to exist in internal regions of proteins, outside of traditional N-terminal signal sequences. For example, a single mutation (N184Q or N250Q) in the protein hFasLECD can enhance the level of protein secretion when expressed in *P. pastoris* [10], [11]. A study on a methyl parathion hydrolase OPHC2 (GenBank No. CAE53631) exogenously expressed in *P. pastoris* showed high expression levels (∼5.5 g/L) using 3 L high-cell-density fermentation [12]; however, another methyl parathion hydrolase (MPH, GenBank No. ACC63894), which shares 46% sequence identity with OPHC2, was not secreted into the culture supernatant. These results suggest that internal protein sequences may contain signals that affect secretion. Therefore, these sequences may be used to predict the likelihood of protein secretion when exogenously expressed in *P. pastoris*.

In this study, we propose the Presep method (Predicting the propensity of a protein being secreted into the supernatant when expressed in *P. pastoris*) to identify the secretion state of proteins in *P. pastoris* based on the ensemble learning method random forests (RF). A dataset (Secreprot) was constructed, containing 136 positive proteins experimentally shown to be secreted into the supernatant upon expression in *P. pastoris*, along with 957 negative samples. A pseudo amino acid composition (PseAAC) method was exploited to encode these proteins. Both the predicted and experimental results showed that Presep was an effective classifier for predicting the secretion propensity of a given protein. This method can be used to predict and optimise the secretion possibility of a given protein prior to heterologous expression in *P. pastoris*.

## Results and Discussion

### Training and Validation

**Figure 1:**
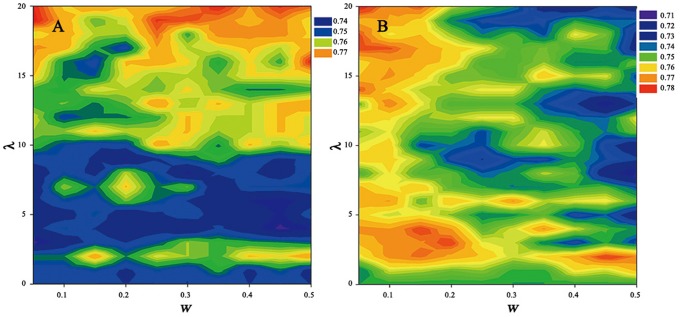
The effect of parameter settings on the prediction performance of Presep for Type I (A) and Type II (B) PseAAC modes. The *x*-axis represents the weight factor (*w*), and the *y*-axis represents the lambda parameter (*λ*). Colour changes indicate differences in the Matthews correlation coefficient (MCC).

To train the models used for Presep, we constructed the Secreprot dataset containing 1093 proteins experimentally validated in *P. pastoris*. To generate a representative set of protein sequences that could accurately identify proteins secreted into the supernatant, we investigated the prediction performance of Type I and Type II PseAAC, respectively. Type I PseAAC is a parallel-correlation type analysis that generates 20+ *λ* discrete numbers to represent a protein [13]. Type II PseAAC is a series-correlation type analysis that generates 20+ *i* * *λ* discrete numbers to represent a protein, with *i* defined as the number of amino acid attributes selected. The parameter of *λ* denoted the correlation rank of amino acids along a protein sequence, which can reflects the rank of correlation and is a non-Negative integer. [14]. Type I and Type II PseAAC models were generated using PseAAC-Builder [15] with different parameters selected for each analysis; the prediction performance for each of these methods is shown in Figure 1. Using a 20-fold cross validation, this method displays a high degree of accuracy for both strategies, with MCC and overall accuracy (Q2) scores of 0.78 and 95%, respectively. However, the parameters used in these analyses, *w* and *λ*, had remarkably different effects on model performance depending on the method used. Using the Type I encoding strategy, w exhibited a much weaker effect on model performance than *λ*. This effect was not seen with the Type II encoding method, with *w* greatly affecting model performance. These results highlight the need to optimize parameter settings based on the encoding method used. The top 10 parameter settings identified in this analysis are shown in Table 1.

**Table 1:** Prediction performance of Presep with different parameters.

Coding Type	*w*	λ	MCC^a^	Q2^b^	Sensitivity	Specificity
I	0.05	19	0.78	0.95	0.82	0.97
II	0.05	20	0.78	0.95	0.78	0.98
I	0.05	20	0.78	0.95	0.82	0.97
I	0.35	20	0.78	0.95	0.82	0.97
I	0.5	16	0.78	0.95	0.82	0.97
II	0.45	2	0.78	0.95	0.83	0.97
I	0.25	19	0.77	0.95	0.81	0.97
I	0.45	20	0.77	0.95	0.81	0.97
II	0.15	4	0.77	0.95	0.82	0.97
II	0.2	3	0.77	0.95	0.82	0.97

Results are based on the Secreprot dataset with 20-fold cross validation.

a MCC, Matthews correlation coefficient.

b Overall prediction accuracy.

### Prediction Performance of Presep

Receiver operating characteristics (ROC) scores are often used as the primary measure to gauge the performance of machine-learning methods and provide an overview of possible cut-off levels [16]. The ROC scores of the random classifier and Presep classifier are shown in Figure S1. The area under the curve with the best parameters of the two encoding schemes was 0.94. This result clearly demonstrates that the Presep classifier was not a random predictor, and could efficiently distinguish between soluble proteins and inclusion body proteins.

**Figure 2:**
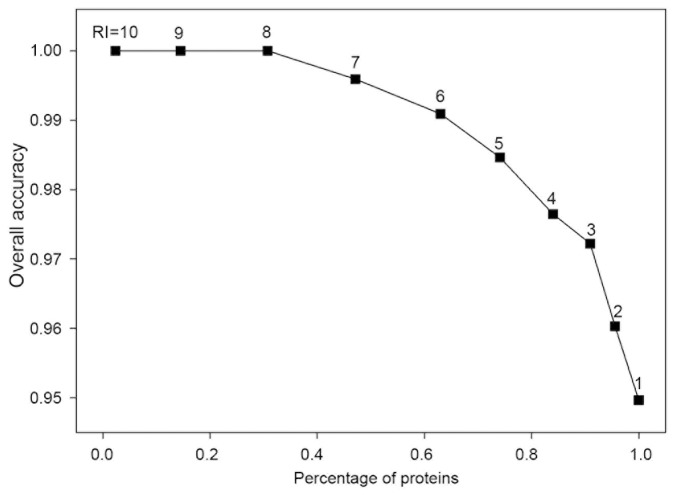
The average prediction accuracy calculated cumulatively with RI above a given value. This result was obtained using RF with a 20-fold cross validation.

When machine-learning approaches are used to classify samples, it is important to know the reliability of the prediction result [17], [18], [19]. In this study, a reliability index (RI) ranging from 0 to 1 was assigned to a predicted protein based on the RF output. Provided that an output of RF for a protein is *O*, the value of RI is computed as RI = INTEGER (20×bsolute [*O*–0.5]). The closer the prediction output is to 1, the greater the chance of that protein being secreted into the supernatant when expressed in *P. pastoris*. Conversely, the closer the prediction score is to 0, the lower the chance that protein will be secreted into the supernatant. The RI value provides a rough measure of certainty for a given classification, and therefore may be used as an indicator of prediction certainty for a particular protein. Figure 2 shows the expected prediction accuracies along with the fraction of proteins with a given RI value. For example, approximately 74% of the proteins obtained an RI ≥ 5, and of these 98% were predicted correctly. This result was obtained using RF with a 20-fold cross validation.

### Terminal Effect on the Secretion of the Target Protein

**Figure 3:**
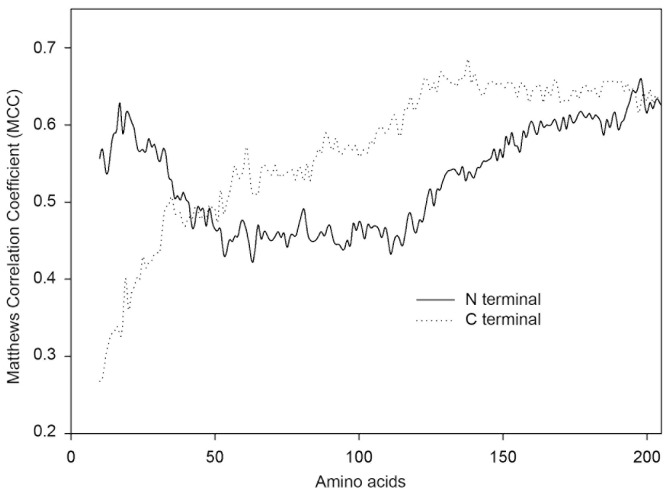
Prediction performance of Presep with different protein lengths. The *x* axis represents the selected residues at N or C terminal that were used to predict the secretion propensity in the dataset. The *y* axis represents the prediction performance, which was evaluated by Matthews correlation coefficient (MCC). A Type I PseAAC coding scheme was used with a weight factor (w) of 0.05 and a lambda parameter (λ) of 19. Results are based on the Secreprot dataset with 20-fold cross validation.

Different protein lengths, measured from either the N- or C-terminal, were used to test classification performance. As shown in Figure 3, significant differences in prediction performance were seen between the N- and C-terminal sequences. The classifier obtained a high degree of accuracy using 17 amino acids at the N-terminal, not including the signal peptide. In contrast, the prediction accuracy using short C-terminal sequences was very low. These results indicate a greater degree of N-terminal sequence variability between secretion-positive and secretion-negative proteins, suggesting that N-terminal sequences may be more important for protein secretion than equivalent regions from the C-terminal.

### Experimental Results

We used β-galactosidase as a reporter to test the prediction performance of Presep. Six galactosidase genes were used to verify prediction performance. Two β-galactosidase genes, LacB from *Aspergillus candidus* and BglKL from *Kluyveromyces lactic,* were isolated from eukaryotes; the remaining four genes were isolated from bacteria. Among the four bacterial strains, three genes were isolated from Gram-positive strains (BglZQ, GalC168, and BG42–106) and one (CelB) was from a Gram-negative strain. Three genes (CelB, BglZQ and GalC168) were from aerobic bacteria, and one gene (BG42–106) was from anaerobic bacteria.

**Figure 4:**
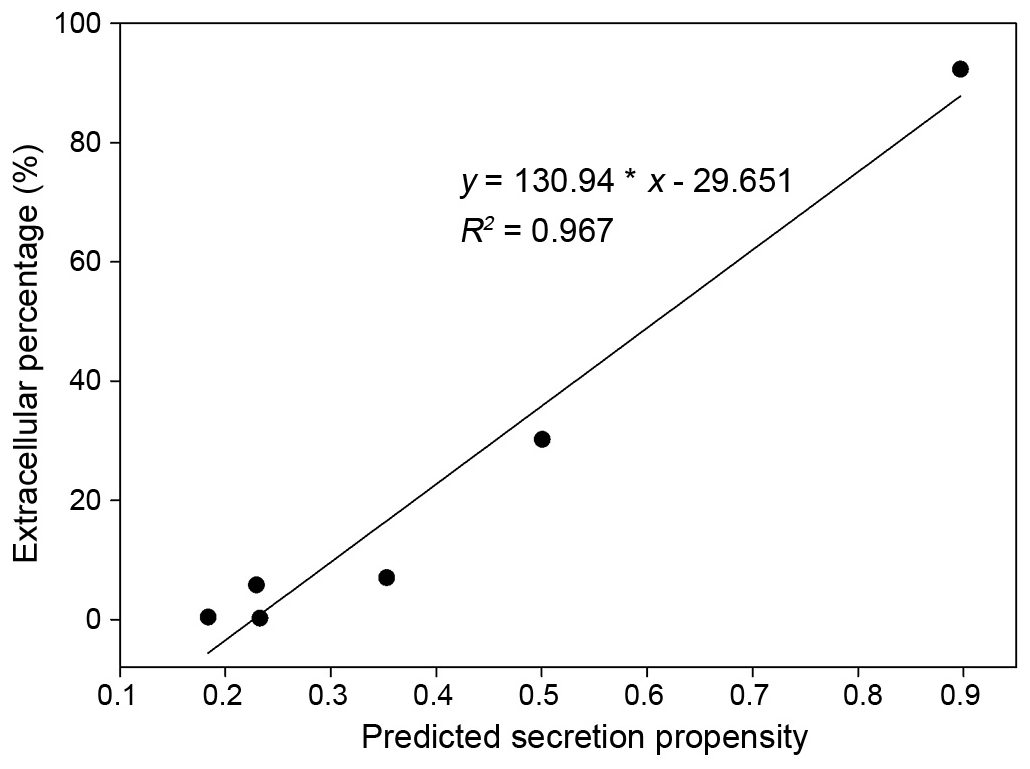
Correlation between the predicted secretion propensity, as determined by Presep, and the extracellular percentage (%) determined using the experimental method.

For these constructs, protein secretion levels were quantified as a percentage of extracellular activity relative to total β-galactosidase activity. The Presep model predicted a high likelihood of secretion for LacB; this result was confirmed by experimental analyses, with LacB showing the highest secretion percentage (92.3%) among all β-galactosidases tested (Table 2). The three β-galactosidases with low predicted secretion propensities (CelB, BglZQ and GalC168) were also validated experimentally, with very low β-galactosidase activity detected in culture media. Overall, the predicted secretion propensities for all six constructs tested were highly correlated with secretion percentage (R^2^ = 0.967; Figure 4).

**Table 2:** Predicted propensity and the experimental results on the six β-galactosidase.

	Predicted propensity		Experiment (%)	
Protein	No secretion	Secretion	Intracellular	Extracellular
LacB	0.10	0.90	7.7±1.3	92.3±0.3
CelB	0.65	0.35	93.0±2.2	7.0±2.2
BglKL	0.50	0.50	69.8±3.3	30.2±3.3
BglZQ	0.82	0.18	99.6±0.1	0.4±0.1
GalC168	0.77	0.23	94.2±3.8	5.8±3.8
BG42–106	0.77	0.23	99.8±0.1	0.2±0.1

The coding scheme of six β-galactosidase proteins using the Type I PseAAC mode, with a weight factor (*w*) of 0.05 and a lambda parameter (*λ*) of 19.

In addition to the examples of β-galactosidase, we also predicted the secretion propensity of the two methyl parathion hydrolases, which the sequence identity of the two proteins is 46%. The predicted secretion propensities of the two proteins, OPCH2 and MPH were 0.68 and 0.48, respectively. The results indicated that the protein OPCH2 could secret, but MPH is difficult to secret in *Pichia pastoris*. The predicted results are consisting with the experimental results. All of the results indicate that internal protein sequences contain detectable signals that may affect the protein secretion in *P. pastoris*, consistent with the Presep model hypothesis.

The evidence presented here demonstrates the utility of the Presep model for predicting protein secretion propensity. However, more work is necessary to identify the sequence factors affecting protein secretion, and to develop protein design methods to improve secretion efficiency. Such work will require characterization of additional proteins with known secretion percentages, along with development of advanced machine-learning methods to better understand the factors influencing protein secretion.

## Materials and Methods

### Datasets

We constructed the Secreprot dataset to train the model and test the robustness of Presep. All proteins secreted into the supernatant upon expression in *P. pastoris* were defined as positive samples; all other proteins were defined as negative samples. Positive samples were collected in three steps. First, related papers with the words “*Pichia pastoris*, express, and supernatant” in the abstract, title, or key words were selected from the Web of Science, with a cut-off date of August 8, 2012; a total of 1080 papers were identified. From these, we selected papers that successfully demonstrated expression of foreign genes in *P. pastoris*, along with secretion of these proteins into the culture supernatant; the accession numbers of these genes were found in each of these papers. Furthermore, the secretion of the protein needed to be independent of secretion-enhancing fusion tags or chaperone co-expression. These criteria were used to ensure that the observed secretion in *P. pastoris* was due to its own sequence features rather than sequence-independent factors. Finally, sequences for each of these proteins were downloaded from the National Center for Biotechnology Information (NCBI). From this analysis, a total of 136 proteins were identified; these proteins were used to define the secretion-positive samples in the Secreprot dataset.

Negative samples were difficult to collect from the published reports, as publication of negative data is rare. However, many studies have described proteins that are not secreted in the native host, and are therefore difficult to secrete in *P. pastoris* [20], [21]. Therefore, we collected sequences of proteins from the PSORT database that was experimentally proven to be expressed only in cytoplasm [22], [23] to construct a large dataset of secretion-negative samples.

Proteins that encode one or more transmembrane helices were removed from the dataset, as membrane proteins contain distinct sequence features not generally found in soluble proteins. To avoid any bias conferred by homologous sequences, the CD-HIT tool [24], [25], [26], [27] was used to remove sequences exhibiting >80% sequence identity. Signal peptides were removed using the software SignalP 4.0 [28]; short proteins of <50 amino acids were also excluded. As a result, the final Secreprot dataset consisted of 1093 proteins, including 136 positive and 957 negative samples. These proteins can be downloaded from the Presep website at http://www.mobioinfor.cn/Presep.

### Random Forests

Random forests (RF) is an ensemble machine-learning methodology introduced by Leo Breiman [29]. The basic idea of ensemble learning is to boost the performance of a number of weak learners by means of a voting scheme, where a weak learner can be an individual decision tree, a single perceptron/sigmoid function, or another simple and fast classifier [30]. Moreover, RF does not require optimization of a large number of parameters. Here, the RF algorithm was run in the R programming environment (http://www.r-project.org/).

### Protein Encoding Schemes

To develop a classification model of RF, each protein sequence in the training dataset should be encoded by a feature vector. In the present study, we attempted to use the PseAAC of proteins to predict the propensity of a given protein being secreted into the supernatant when expressed in *P. pastoris* [31], [32]. The software PseAAC-Builder was used to transform protein sequences of variable length into fixed-length feature vectors [15]. Three different parameters can be used to generate distinct PseAAC outputs. Six physicochemical characteristics of amino acids, hydrophobicity, hydrophilicity, side chain mass, pKa of the α-COOH group, pK of the α-NH^3+^ group, and pI at 25°C, are employed to calculate the correlations between amino acids at different positions along the protein sequence, which the values of the six physicochemical characteristics of amino acids were shown in the Table S2.The resulting dimension is (20+ *λ*) for a type I PseAAC output and (20+ *i* * *λ*) for a type II output, where *λ* and *i* denote the correlation rank of amino acids along a protein sequence and the number of amino acid characters, respectively.

### Assessment of Prediction System

The performance of the method was assessed based on a 20-fold cross validation. True positives (TPs) and true negatives (TNs) were identified as positive and negative samples, respectively. False positives (FPs) were secretion-negative samples incorrectly identified as positive; false negatives (FNs) were secretion-positive samples incorrectly identified as negative. Prediction performance was tested for sensitivity (TP/(TP+FN)), specificity (TN/(TN+FP)), and overall accuracy (Q2), and quantified using the Matthews correlation coefficient (MCC). Q2 and MCC values were calculated as follows:
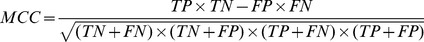
(1)

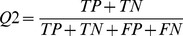
(2)


### Experimental Verification

For direct experimental verification of our predictions, six β-galactosidase genes (LacB, CelB, BglZQ, BglKL, GalC168 and BG42–106) were expressed in *P. pastoris* (Table S1). Each of these genes was cloned and inserted into the pPIC9 vector (Invitrogen, USA) to generate recombinant constructs, cloned into Escherichia coli Trans1-T1™ (Transgen, China), and then expressed in *P. pastoris* strain GS115 (Invitrogen) according to the manufacturer’s instructions. Recombinant genes were inserted downstream of the α-mating factor signal of vector pPIC9, and its expression was controlled by the AOX1 promoter (Figure S2). In addition, the inserted β-galactosidase gene was without its original signal peptide. Transformed cells were plated onto RDB plates and incubated at 30°C for 2–3 days until colonies appeared. Twenty-four positive *P. pastoris* transformants for each recombinant construct were randomly selected, according to the manufacturer’s instructions. Each positive clone was transferred into 20 mL BMGY medium and cultivated at 30°C in an orbital shaker at 200 rpm for 48 h. Cells were pelleted by centrifugation at 5,000× g for 5 min, suspended in 10 mL BMMY medium (containing 0.5% methanol), and then cultured at 30°C for another 48 h (methanol was added every 12 h at a concentration of 0.5%); then cell density was measured based on absorbance at 600 nm. Next, the culture was centrifuged and the medium supernatant was collected to detect extracellular β-galactosidase activity, determined as described previously [33]. Pelleted cells were frozen in liquid nitrogen and ground into a fine powder, then suspended in the appropriate pH buffer. After centrifuged at 5,000× g for 5 min, the supernatant was used to detect intracellular β-galactosidase activity. Total protein concentration was calculated using a protein assay kit (Bio-Rad). For each recombinant *P. pastoris* strain, the protein secretion level was defined as the level of extracellular β-galactosidase activity relative to total β-galactosidase activity (defined as the sum of all extracellular and intracellular activity).

## Supporting Information

Figure S1 **ROC curves of random prediction and Presep prediction with two different parameters.** The parameter 1 means that w is 0.05, λ is 19 and the type I coding scheme. The parameter 2 means that w is 0.05, λ is 20 and the type II coding scheme. The ROC curves were obtained using Random forests with the 20-fold cross validation test on the Secreprot dataset.
(DOC)

Figure S2 **Schematic diagram of the recombinant constructs.**
(DOC)

Table S1 **Predicted propensity and the experimental results on the six β-galactosidases.**
(DOC)

Table S2 **The hydrophobicity, hydrophilicity, mass, pK1(alpha-COOH), pK2(NH3) and pI(at 25°C) values.**
(DOC)
