# Supplementary material for: Presep: Predicting the Propensity of a Protein Being Secreted into the Supernatant when Expressed in Pichia pastoris
Source: PLoS One. 2013 Nov 21;8(11):e79749. doi: 10.1371/journal.pone.0079749 (PMC3836778; doi:10.1371/journal.pone.0079749)

Figure S1. ROC curves of random prediction and Presep prediction with two different parameters. The parameter 1 means that *w* is 0.05, *λ* is 19 and the type I coding scheme. The parameter 2 means that *w* is 0.05, *λ* is 20 and the type II coding scheme. The ROC curves were obtained using Random forests with the 20-fold cross validation test on the Secreprot dataset.


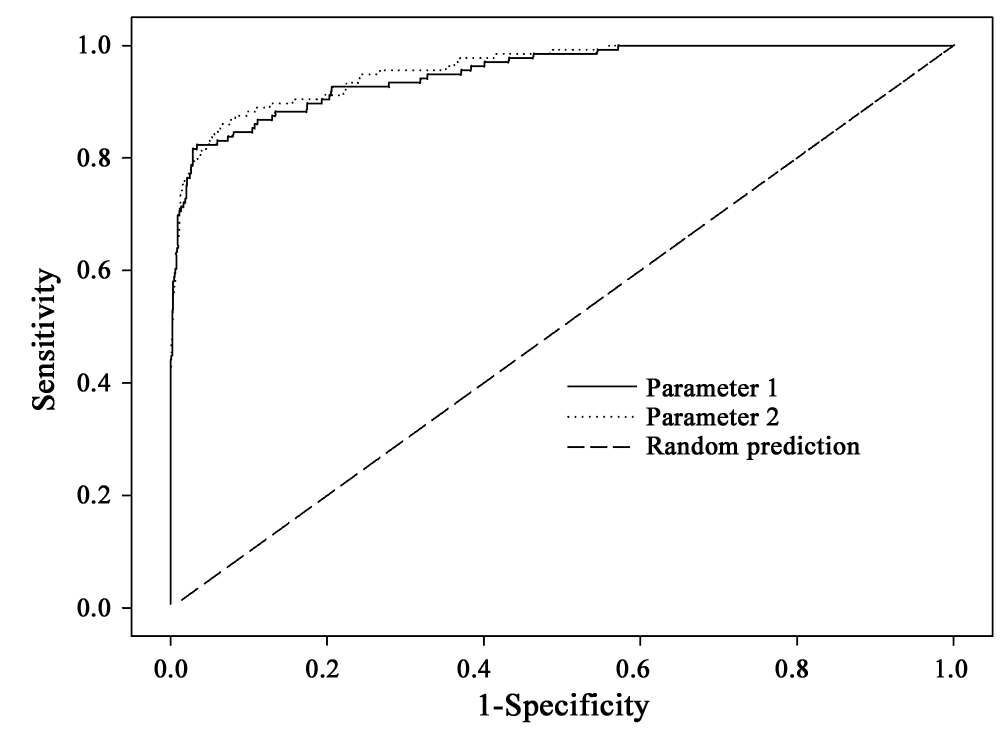

Supplement: Figure S1 — ROC curves of random prediction and Presep prediction with two different parameters. The parameter 1 means that w is 0.05, λ is 19 and the type I coding scheme. The parameter 2 means that w is 0.05, λ is 20 and the type II coding scheme. The ROC curves were obtained using Random forests with the 20-fold cross validation test on the Secreprot dataset. (DOC) [file pone.0079749.s001.doc]
