# Supplementary material for: Presep: Predicting the Propensity of a Protein Being Secreted into the Supernatant when Expressed in Pichia pastoris
Source: PLoS One. 2013 Nov 21;8(11):e79749. doi: 10.1371/journal.pone.0079749 (PMC3836778; doi:10.1371/journal.pone.0079749)
Supplement: Figure S2 — Schematic diagram of the recombinant constructs. (DOC) [file pone.0079749.s002.doc]

Figure S2. Schematic diagram of the recombinant constructs


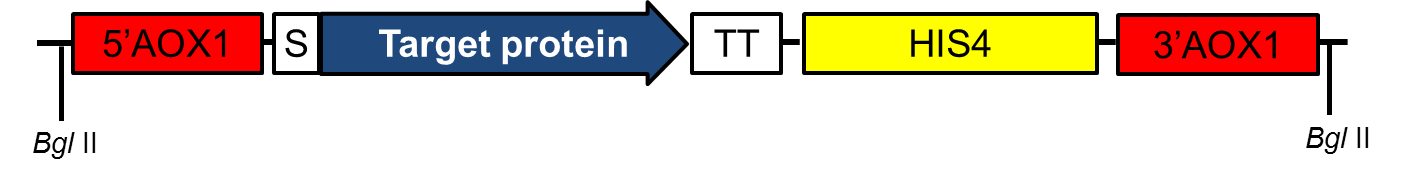


Abbreviations: 5’AOX1, 5’*AOX1* promoter fragment; S, α-Factor secretion signal; TT, 3’*AOX1* transcription termination; HIS4, *HIS4* ORF; 3’AOX1, 3’*AOX1* fragment.
