# Supplementary material for: Presep: Predicting the Propensity of a Protein Being Secreted into the Supernatant when Expressed in Pichia pastoris
Source: PLoS One. 2013 Nov 21;8(11):e79749. doi: 10.1371/journal.pone.0079749 (PMC3836778; doi:10.1371/journal.pone.0079749)
Supplement: Table S1 — Predicted propensity and the experimental results on the six β-galactosidases. (DOC) [file pone.0079749.s003.doc]

**Table S1. Predicted propensity and the experimental results on the six β-galactosidases**

| Protein | GenBank No. | Origin | Predicted propensity | | Reliability Index | Experiment | |
| --- | --- | --- | --- | --- | --- | --- | --- |
|  |  |  | No secretion | Secretion |  | Intracellular (%) | Extracellular (%) |
| LacB | CAD24293 | *Aspergillus candidus* | 0.10 | 0.90 | 7 | 7.7 ± 1.3 | 92.3 ± .3 |
| CelB | KF420204 | *Pyrococcus furiosus* | 0.65 | 0.35 | 2 | 93.0 ± 2.2 | 7.0 ± 2.2 |
| BglKL | KF420203 | *Kluyveromyces lactic* | 0.50 | 0.50 | 0 | 69.8 ± 3.3 | 30.2 ± 3.3 |
| BglZQ | KF420202 | *Bacillus licheniformis* | 0.82 | 0.18 | 6 | 99.6 ± 0.1 | 0.4 ± 0.1 |
| GalC168 | FJ906971 | *Cellulomonas sp*. | 0.77 | 0.23 | 5 | 94.2 ± 3.8 | 5.8 ± 3.8 |
| BG42–106 | JX188444 | *Bifidobacterium animalis* | 0.77 | 0.23 | 5 | 99.8 ± 0.1 | 0.2 ± 0.1 |

The coding scheme of six β-galactosidase proteins using the Type I PseAAC mode, with a weight factor (*w*) of 0.05 and a lambda parameter (*λ*) of 19.
