# Supplementary material for: Presep: Predicting the Propensity of a Protein Being Secreted into the Supernatant when Expressed in Pichia pastoris
Source: PLoS One. 2013 Nov 21;8(11):e79749. doi: 10.1371/journal.pone.0079749 (PMC3836778; doi:10.1371/journal.pone.0079749)
Supplement: Table S2 — The hydrophobicity, hydrophilicity, mass, pK1(alpha-COOH), pK2(NH3) and pI(at 25°C) values. (DOC) [file pone.0079749.s004.doc]

**Table S2. The hydrophobicity, hydrophilicity, mass, pK1(alpha-COOH), pK2(NH3) and pI(at 25ºC) values**

| Amino acid | Hydrophobicity ^a^ | Hydrophilicityy^b^ | Mass ^c^ | pK1(a-CO2H) ^d^ | pK2(NH3) ^d^ | pI(at 25oC) ^d^ |
| --- | --- | --- | --- | --- | --- | --- |
| A | 0.62 | -0.5 | 15 | 2.35 | 9.87 | 6.11 |
| C | 0.29 | -1 | 47 | 1.71 | 10.78 | 5.02 |
| D | -0.9 | 3 | 59 | 1.88 | 9.6 | 2.98 |
| E | -0.74 | 3 | 73 | 2.19 | 9.67 | 3.08 |
| F | 1.19 | -2.5 | 91 | 2.58 | 9.24 | 5.91 |
| G | 0.48 | 0 | 1 | 2.34 | 9.6 | 6.06 |
| H | -0.4 | -0.5 | 82 | 1.78 | 8.97 | 7.64 |
| I | 1.38 | -1.8 | 57 | 2.32 | 9.76 | 6.04 |
| K | -1.5 | 3 | 73 | 2.2 | 8.9 | 9.47 |
| L | 1.06 | -1.8 | 57 | 2.36 | 9.6 | 6.04 |
| M | 0.64 | -1.3 | 75 | 2.28 | 9.21 | 5.74 |
| N | -0.78 | 0.2 | 58 | 2.18 | 9.09 | 10.76 |
| P | 0.12 | 0 | 42 | 1.99 | 10.6 | 6.3 |
| Q | -0.85 | 0.2 | 72 | 2.17 | 9.13 | 5.65 |
| R | -2.53 | 3 | 101 | 2.18 | 9.09 | 10.76 |
| S | -0.18 | 0.3 | 31 | 2.21 | 9.15 | 5.68 |
| T | -0.05 | -0.4 | 45 | 2.15 | 9.12 | 5.6 |
| V | 1.08 | -1.5 | 43 | 2.29 | 9.74 | 6.02 |
| W | 0.81 | -3.4 | 130 | 2.38 | 9.39 | 5.88 |
| Y | 0.26 | -2.3 | 107 | 2.2 | 9.11 | 5.63 |

**a The hydrophobicity values are from JACS, 1962, 84: 4240-4246. (C. Tanford).**

**b The hydrophilicity values are from PNAS, 1981, 78:3824-3828 (T.P.Hopp & K.R.Woods).**

**c The side-chain mass for each of the 20 amino acids.**

**d CRC Handbook of Chemistry and Physics, 66th ed., CRC Press, Boca Raton, Florida (1985).**
